# Simultaneous Measurement of Zinc, Copper, Lead and Cadmium in Baby Weaning Food and Powder Milk by DPASV 

**Published:** 2014

**Authors:** Naficeh Sadeghi, Mohammad Reza Oveisi, Behrooz Jannat, Mannan Hajimahmoodi, Abdolazim Behfar, Masoomeh Behzad, Narges Norouzi, Morvarid Oveisi, Behzad Jannat

**Affiliations:** a *Department of Drug and Food Control, School of Pharmacy, Tehran University of Medical Sciences, Tehran, Iran. *; b *Water Safety Research Center, Food and Drug Organization, Ministry of Health and Medical Education, Tehran, Iran. *; c *Halal Research Center,food and Drug Organization, Ministry of Health and Medical Education, Tehran, Iran. *; d *Food Sciences and Medical Hydrology Department, School of Pharmacy, Ahvaz Jundishapur University of Medical Sciences, Ahvaz,Iran. *; e *Azad University,Tehran, Iran. *

**Keywords:** Zinc, Copper, Lead, Cadmium, Baby weaning food, Powder milk, Voltammetry

## Abstract

Apart from the breast milk, infant formula and baby weaning food have a special role in infant diet. Infants and young children are very susceptible to amount of trace elements. Copper and zinc are two elements that add in infant food. Lead and cadmium are heavy metals that enter to food chain unavoidably. DPASV is a benefit and applicable method for measurement of trace elements in food products. In this study, concentration of zinc, copper, lead and cadmium in four brands of baby food (rice and wheat based) and powder milk was analyzed with DPASV and polarograph set. Total Mean ± SE of zinc, copper, lead and cadmium in baby foods (n = 240) were 11.86 ± 1.474 mg/100g, 508.197 ± 83.154 μg/100g, 0.445 ± 0.006, 0.050 ± 0.005 mg/Kg respectively. Also these amount in powder milk (n = 240) were 3.621± 0.529 mg/100g, 403.822 ± 133.953 μg/100g, 0.007 ± 0.003, 0.060 ± 0.040 mg/Kg respectively. Zinc level in baby food type I was higher than lablled value (P = 0.030), but in other brands was not difference. Concentration of copper in all of samples was in labeled range (P > 0.05). In each four products, level of lead and cadmium were lower than the standard limit (P < 0.05). Amount of zinc and lead in baby food I, had difference versus other products. Concentration of zinc, camium in baby food type I, was higher than type II (P = 0.043, 0.001 respectively). Concentration of lead and cadmium in baby food type II, was higher than infant formulas, but are in standard limit.

## Introduction

Human milk has the most suitable capability to meet infant and young children᾽s necessities in the first six months of life (1, 2, 3, 4 and 5). After these ages, nutritional and psychological needs of infant are increased and breast milk isn᾽t enough. So complementary or weaning food is recommended by WHO (6). Weaning is passing from exclusive milk feeding to family food and is a very vulnerable period (7). Infant and children are very susceptible and vulnerable to the whole amount of food᾽s elements, and their effects on health .They have a low ability in excretion processes and immunity (8). Zinc and copper are two essential elements in human body. Zinc deficiency can cause to decrease immune system delayed in growth and hypogonadism. Excess of zinc results in nausea, vomiting and headache. Either copper deficiency, accompanied by failures in collagen, elastin reticulation and problems in tissues,especially in arteries. It᾽s excess can cause to diarrhea, nausea, vomiting, cirrhosis and anemia (6, 9, 10). Some of heavy metals like lead and cadmium enter in the food᾽s chain naturally and it is unavoidable (11). Infants and young children are the most risky group to these metals (8). Lead is a known neurotoxin for infant, and can cause to reduced IQ, learning disabilities and irreversibly effects development of the nervous system. Cadmium leads to kidneys disfunction and has estrogenic propreties (12). International organization such as Unicef 1999, emphasise on control and assessment of babies᾽food product᾽s by purpose of healthy (8). Differential pulse voltammetry has been widely used for trace metal measurement in food samples (13). DPASV, it makes possible to determine simultaneously several trace elements and both thermodynamic and kinetic assays (14, 15). ASV has been widely used for trace metal measurement in food samples (16). This method is inexpensive, selective and sensitive (14).

## Experimental

*Apparatus*

The polarograph set was Trace analyzer model 746 instruments (Metrohm AG Ltd.Switzerland). Its glassy cell has three electrode, HMDE as the working electrode, axillary electrode from platinum and Ag/AgCl electrode as the reference electrode.

*Reagents*

The chemicals, tartaric acid, CH3COONa, Pb(NO3)2, Cd(NO3)2, Cu(NO3)2, Zn(NO3)2 and HNO3 in analytical grades and were purchased from Merck Company Germany. The metal stock solution (1 g/l) was prepared in 0.005 molar HNO3. The acetate buffer (0.2 M, pH = 4.7) containing 0.2 M tartaric acid, which serve as a supporting electrolyte. A number of 120 samples of each baby foods and formulas were prepared from market Tehran, Iran.

*Sample preparation*

A sample weighted 2 g among with 5 mL nitric acid 65% were transferred into a cleaned glass vessel. The sample was evaporated to dryness on a hot plate. Temperature of the hot plate was gradually increased from low to maximum over a period of 1-2 h. The charred sample was transferred to a muffle furnace and ashed at 450-500 oC for 24 h. The residue was wet with 1 mL nitric acid 65% and 9 mL distilled water, and then crucible was heated on a hot plate near boiling to complete dissolution of the residue. The sample was then quantitatively transferred to a 25 mL volumetric flask and diluted to volume. 

The complex composition of the sample may cause to changes in the calibration slopes and, for this reason, the elements᾽ concentration was determined using the standard addition technique with three addition of the standard stock solution. At the first with 10 mL acetate buffer the blank polarogram of the supporting electrolyte was first recorded. Next, 500 μL of unknown solution were added in cell and the polarogram done. 100 μL of standard solution added in the cell and the polarogram was recorded. This operation was done three times. The concentrations were calculated after the second standard addition using linear regression method. In the application of stripping method for food analysis, the major challenge is often the sample pretreatment, which can interfere with the organic matrix, which can interfere with the electrochemical processes (17).

## Results

In the present study the level of zinc, copper, lead and cadmium was determined in the some brands of baby weaning food and infant formulas. A number of 120 samples of each brand were prepared from market Tehran, Iran. All of measurements of trace elements with DPASV method were repeated three times. Obtained concentrations from samples were compared with reference values. These calculating were done by SPSS and data was analyzed with ANOVA and Tukey tests. The mean concentrations of zinc and copper and reference values are shown in Table 1 and 2. Comparison between means of elements᾽ concentration in baby food and powder milk with t-test for equality of mean. 

**Table 1:** Analysis of some elements in baby weaning foods

**Babyweaning food**	**Zn ( mg/100g)** **Mean ± SE**	**Cu ( μg/100g)** **Mean ± SE**	**Pb ( mg/Kg)** **Mean± SE**	**Cd ( mg/Kg)** **Mean ± SE**
Type I (n = 120)	14.83 **± **2.30 *	586.40**± **89.46	0.07 **± **0.01 *	0.06 **± **0.00 *
Type II (n =120)	8.90 **± **1.70	430.00**±**77.00	0.02**± **0.00	0.03 **± **0.00
Total (n = 240)	11.86 **± **1.47	508.19**±**83.15	0.445±0.006	0.05±0.005
labeled value Standard limit	7.00	400.00	0.20	0.20

* P < 0.05

**Table 2:** Analysis of some elements in infant formulas

**Infant formula**	**Zn (mg/100 g)** **Mean ± SE**	**Cu (μg/100 g)** **Mean ± SE**	**Pb (mg/Kg)** **Mean ± SE**	**Cd (mg/Kg)** **Mean ± SE**
A ( n = 120)	4.00 **± **0.77	400.00**±**122.20	0.01 **± **0.00	0.08 **± **0.06
B (n =120)	3.300 **± **0.30	409.00**±**146.00	0.05 ± 0.00 *	0.04 **± **0.02
Total ( n = 240)	3.62 **± **0.52	403.82**±**133.95	0.007±0.003	0.06 **± **0.04 *
Lablled valueStandard limit	3.00	400.00	0.20	0.15

* P < 0.05

## Discussion

A number of two hundred and forty baby weaning food and infant formula samples were analyzed for zinc, copper, lead and cadmium. All the results were discussed and compared with the data from their label and proposed guidelines published by WHO / Unicef. Zn, Cu and Pb were determined the highest (14.830 mg/100g, 586.400 μg/100g, 0.069 mg/Kg respectively) in baby weaning food type I. Followed by Cd (0.080 mg/Kg)in infant formula. A. DPASV method which was used for analysis has many advantages. DPASV method needs to few time and low cost to analysis, besides it has high sensitivity, ability to simultaneous determination of elements, versus other methods (18). As seen in Table 1, zinc level in baby weaning food I, is higher than labeled value (P < 0.05), but in other samples there was not difference. In all of samples, copper concentration was in acceptable range with label comparison. The balance amount of essential elements has a key role in the metabolism of protein, carbohydrate and lipid. Low level of essential elements has adverse effect and decrease immunity (19). Zinc and copper are two essential for growth, but excess of them can be harmful. Many brands of infant formula have been designed to provide required nutrients as recommended diet intake (RDI) of minerals for infants (20). Lead and cadmium amounts in baby foods and infant formulas were significantly lower than regulatory or standard levels ( P < 0.01) and the levels of lead and cadmium were lower in comparison to previous our research for infant formula which were significantly more than standard and labeled value (16). Hence, none of the infant formulas, foods exceeded the limit of lead and cadmium in this study. However, the presence of lead and cadmium in infant food is of great concern since infants are particularly sensitive to effects of ingested toxicants. Gastrointestinal absorption of chemicals is elevated prior to weaning while many organs are undeveloped, so infant health goes at risk (21). Exposure to Pb in utero and during infancy irreversibly affects development of the nervous system, causing reduced IQ and learning disabilities (12). Chronic exposure to Cd and Pb is associated with kidney damage (22). Several researchers have studied on heavy elements in baby food. M. Moraes etal in Brazil (2009) analyzed two brands baby food about zinc, copper and iron. Means of copper in brand I and II with FAAS method were higher than submitted ranges. Levels of lead and cadmium in both types of baby food, were lower than standard limit (6). Alkhalifa *et al*. (2010) searched about key elements by ICP-OES in commercially infant formula and baby foods in Saudi Arabia. They found that none essential elements were comparable with the set limit by international pediatric guideline of infant formula. Pb and Cd were detected in almost all samples with mean range of 5 ppm (19). Another study on 437 individual samples of infant formula in Canada shows that Cd and Pb concentrations were all below 0.01 and 0.04 ngg-1, respectively (12). In a study by P.Vinos (Spain, 2000), cadmium was found at very low concentration, while lead was not detected in any samples of baby foods (18). 

With comparing between two types of baby food in our study, zinc, lead and cadmium in type I (wheat) was higher than the type II (rice) baby foods (P < 0.05). This difference may be due to variation in ability of plants intake of these trace element from water, soil and air. Environmental pollution is a human phenomenon. Mineral and industrial activities cause to enter pollutant heavy metals such as lead and cadmium to source water. Different plants such as wheat and rice absorb these elements based on their capacity. In a study, have showed absorbance and accumulation of cadmium in rice plant was 0.0083 mmol/g and for wheat was 0.1032 mmol/g (23). Plants abilityin accumulation of cadmium is different, too (24).

In a study by A. Ikem *et al*. in USA (2002), after analysis of seventeen brands of infant formula, the average levels of Zn, Cu, Pb and Cd in USA soy based powder formula were generally higher than average of element᾽s levels in the milk based in powder from the UK, Nigeria and USA (25). With determination of zinc, copper, lead and cadmium in powder milk products in Iran, concentrations of lead and copper were found higher than the standard and label values (P < 0.001) (Jannat B, 2009) (16). This elements level could be attribute to the different manufacturing practices, variations in quality of raw materials, finished products and packaging containers used by infant formula manufacturers (25). Technical errors could also influence the accuracy and precision of results such as inevitable deterioration in the performance of the equipments as well as the aging of reagents (26). 

Tukey test performance on the results defined that the level of zinc and lead in baby food was higher than infant formula. This result can be caused to by pollution of grains such as wheat and rice. Many elements can be present in foods naturally, or through human activities, such as processing, storage, farming activities and industrial emission. In a similar comparative study in 2010, concentration of lead was in submitted area and cadmium in cereal baby foods was higher than infant formulas (19). It seems that the most reason of entering lead and cadmium in baby food, is water or other ingredients which are consuming in producing processes.

## Conclusion

The result of this study shows that baby weaning food products and infant formula samples in Iran, are adequate in essential elements of zinc and copper, and toxic heavy elements of Cd and Pb are within safety limits. But it seems that there is need to good quality control periodically, in preparation processing of these baby food products.
